# Unraveling the metabolic potential of biocontrol fungi through omics data: a key to enhancing large-scaleapplication strategies: Omics data unveils biocontrol fungi’s metabolic potential

**DOI:** 10.3724/abbs.2024056

**Published:** 2024-04-29

**Authors:** Haolin Yang, Xiuyun Wu, Caiyun Sun, Lushan Wang

**Affiliations:** State Key Laboratory of Microbial Technology Institute of Microbial Technology Shandong University Qingdao 266237 China

**Keywords:** omics, biocontrol fungi, nonribosomal peptides (NRPs), polyketide synthase (PKS), metabolic transformation

## Abstract

Biological control of pests and pathogens has attracted much attention due to its green, safe and effective characteristics. However, it faces the dilemma of insignificant effects in large-scale applications. Therefore, an in-depth exploration of the metabolic potential of biocontrol fungi based on big omics data is crucial for a comprehensive and systematic understanding of the specific modes of action operated by various biocontrol fungi. This article analyzes the preferences for extracellular carbon and nitrogen source degradation, secondary metabolites (nonribosomal peptides, polyketide synthases) and their product characteristics and the conversion relationship between extracellular primary metabolism and intracellular secondary metabolism for eight different filamentous fungi with characteristics appropriate for the biological control of bacterial pathogens and phytopathogenic nematodes. Further clarification is provided that *Paecilomyces lilacinus*, encoding a large number of hydrolase enzymes capable of degrading pathogen protection barrier, can be directly applied in the field as a predatory biocontrol fungus, whereas *Trichoderma*, as an antibiosis-active biocontrol control fungus, can form dominant strains on preferred substrates and produce a large number of secondary metabolites to achieve antibacterial effects. By clarifying the levels of biological control achievable by different biocontrol fungi, we provide a theoretical foundation for their application to cropping habitats.

## Introduction

Biological control is a low-cost and environmentally friendly biotechnology that aims to control crop pests and pathogens by restraining harmful organisms through the application of antagonistic biocontrol microorganisms (operating as a predator or a biocontrol agent exhibiting antibiosis) and their products. According to experts, the global biological control market will reach US$ 8.7 billion by 2022 [1]. However, the antimicrobial mechanisms of various biocontrol microorganisms under practical field conditions are unclear. This can lead to inadequate supplies of the necessary carbon and nitrogen sources for the growth or production of key metabolites by the biocontrol agent after application to the field, making it difficult for them to form dominant strains in the crop environment. Furthermore, limited secondary metabolism can result in only inadequate quantities of antimicrobial substances being produced, greatly weakening the biocontrol ability of the agent [2]. Therefore, it is necessary to further explore the metabolic potential and specific antimicrobial mechanisms of fungal biocontrol microorganisms to accelerate the development of new, green, safe and efficient biocontrol agents for crop pests and pathogens.

High-throughput omics technologies enable the in-depth exploration of microbial metabolic potential at multiple levels and the analysis of microbial industrial application prospects, greatly accelerating the progress of the development of biocontrol agents [3]. Second-generation genome sequencing can rapidly provide a large number of complex and highly repetitive genomic sequences and relevant annotation information [4]. With the emergence of deep learning-based structural prediction tools, such as AlphaFold2 and RoseTTAFold, a massive number of sequences have been transformed into billions of protein structures, further elucidating the relationship between structure and function [5]. Therefore, the study of the metabolic potential of biocontrol fungi based on sequence and structural omics data has become an increasingly interesting and important topic [6].

This review is based on genomics and structural proteomics to analyze the metabolic potential, both intracellularly and extracellularly, of eight biocontrol fungal species, namely, *Aspergillus niger*, *Paecilomyces lilacinus*, *Trichoderma asperellum*, *Trichoderma atrovirens*, *Trichoderma reesei*, *Trichoderma longibrachiatum*, *Trichoderma harzianum* and *Trichoderma virens*, and to elucidate their specific biocontrol types, providing theoretical guidance for the effective agricultural application of these biocontrol fungi.

## Main Extracellular Metabolism of Biocontrol Fungi

### Nitrogen source metabolism

**Figure 1:**
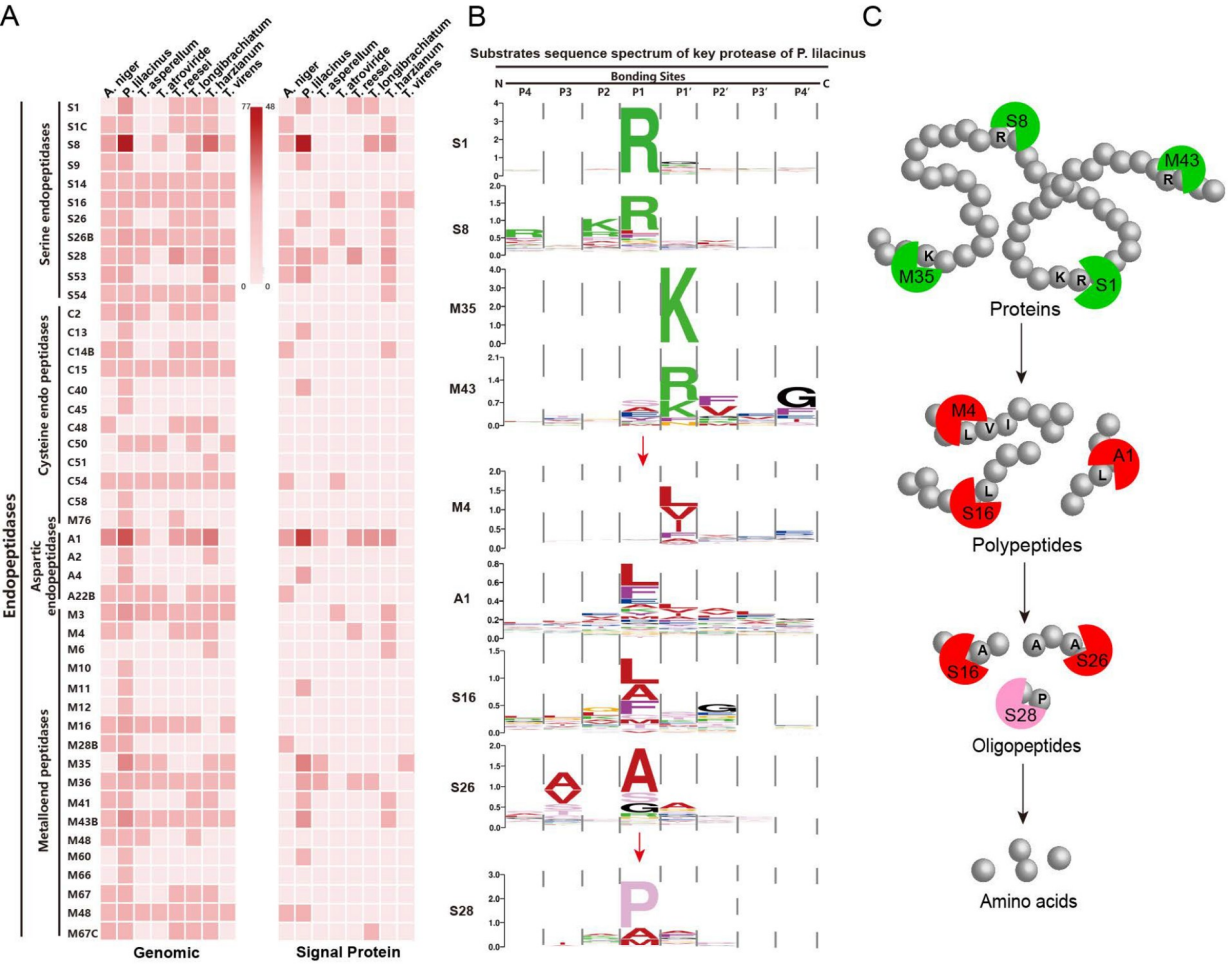
Figure 1 Statistics of protease components in the genome and schematic diagram of the protein degradation model (A) Statistical analysis of protease components in the genomes of 8 biocontrol filamentous fungi. The number of genomes represents the number of proteases in the genome, while the number of signal proteins represents the number of proteases with signal peptides. (B) Substrate sequence spectrum of key proteases in Paecilomyces lilacinus. (C) Schematic diagram of the mode of protein degradation by P. lilacinus.

Filamentous fungi rely on extracellular proteases to degrade proteins and peptides in their habitat to obtain nitrogen sources and to achieve biocontrol effects by disrupting the protective barrier of nematodes [7]. Proteases are generally classified at multiple levels based on the position of the protein chain they hydrolyze, the amino acids and metal ions involved in the catalysis of peptide bond hydrolysis and the sequence similarity of the active structural amino acids [ 8– 10]. Based on these three aspects, the annotated proteases in the eight fungi were classified, and the substrate sequence spectrum was drawn by combining the substrate sequence collections of different families in the MEROPS database to clarify the degradation capabilities of proteases from different biocontrol fungi ( Figure 1).

Generally, protein degradation is initiated by endopeptidases, followed by exopeptidases [11], with a single protease being insufficient to effectively degrade complex proteins as substrates; the degradation of proteins requires the synergistic action of multiple enzymes [12]. The effective degradation process of proteins generally follows a particular process. First, an endopeptidase cleaves the hydrophilic amino acids (Arg, Lys, *etc*.) on the outer surface of the globulin to degrade it into peptones, exposing the hydrophobic amino acids (Leu, Ile, Val, Ala, *etc*.) wrapped inside the protein tertiary structure. A series of proteases that specifically recognize Leu, Ile, Val, Ala and other hydrophobic residues further cleave the peptides produced, and the resulting oligopeptides are transferred into fungal cells via transporters for fungal growth. Most S8 family proteases have been reported to be endopeptidases [13]. The substrate sequence spectrum shows that the S8 protease family of serine endopeptidases specifically recognizes and hydrolyzes the peptide bond after Arg (located on the outer surface of the protein) at the P1 position ( Figure 1B), similar to trypsin. Therefore, the secretion of S8 family proteases plays a key role in the degradation of globulin and the resulting rupture of the nematode body wall [14].

Among the eight filamentous fungi investigated, *P*. *lilacinus* contains the largest number of protease-encoding genes, with a total of 548, which is 4 times greater than that of the other 7 species. Notably, the genes encoding the S8 family of *P*. *lilacinus* underwent significant expansion during evolution, resulting in a total of 77 genes ( Supplementary Table S1). This is one of the important factors that allows *P*. *lilacinus* to directly lyse the body wall of nematodes as prey [14]. In addition to S8 family proteases, *P*. *lilacinus* also contains 11 S26 family proteases that specifically hydrolyze Ala at the P1 position and 21 A1 family proteases that specifically hydrolyze Leu at the P1 position ( Figure 1B). These proteases can further degrade the nematode body wall, cleaving it into peptones and then oligopeptides for *P*. *lilacinus* absorption and utilization. Based on the above analysis of the genome and proteome of *P*. *lilacinus*, a model diagram of its efficient degradation of globulin can be drawn: Proteases such as the S8 and S1 families deconstruct intact globulin, which is then degraded into oligopeptide fragments by proteases, such as the A1, M4, and S28 families, to provide nitrogen to power fungal growth and metabolism ( Figure 1C).

In contrast, the number of proteases carrying signal peptides in the genome of *Trichoderma* typically does not exceed 100, which is less than that of *P*. *lilacinum* ( Supplementary Table S1). *T*. *asperellum*, *T*. *atrovirens*, *T*. *reesei* and *T*. *virens* lack genes encoding the S8 family to open globulin to rupture nematodes [14] but instead secrete more metalloproteinases to degrade small peptides. Through the analysis of omics data, it was found that the composition of this extracellular protease system determines that the ability of *Trichoderma* species to directly prey on nematodes is much lower than that of *P*. *lilacinum*. Compared to other strains, *P*. *lilacinum* has a significant effect on reducing nematodes [15].

### Carbon source metabolism

**Figure 2:**
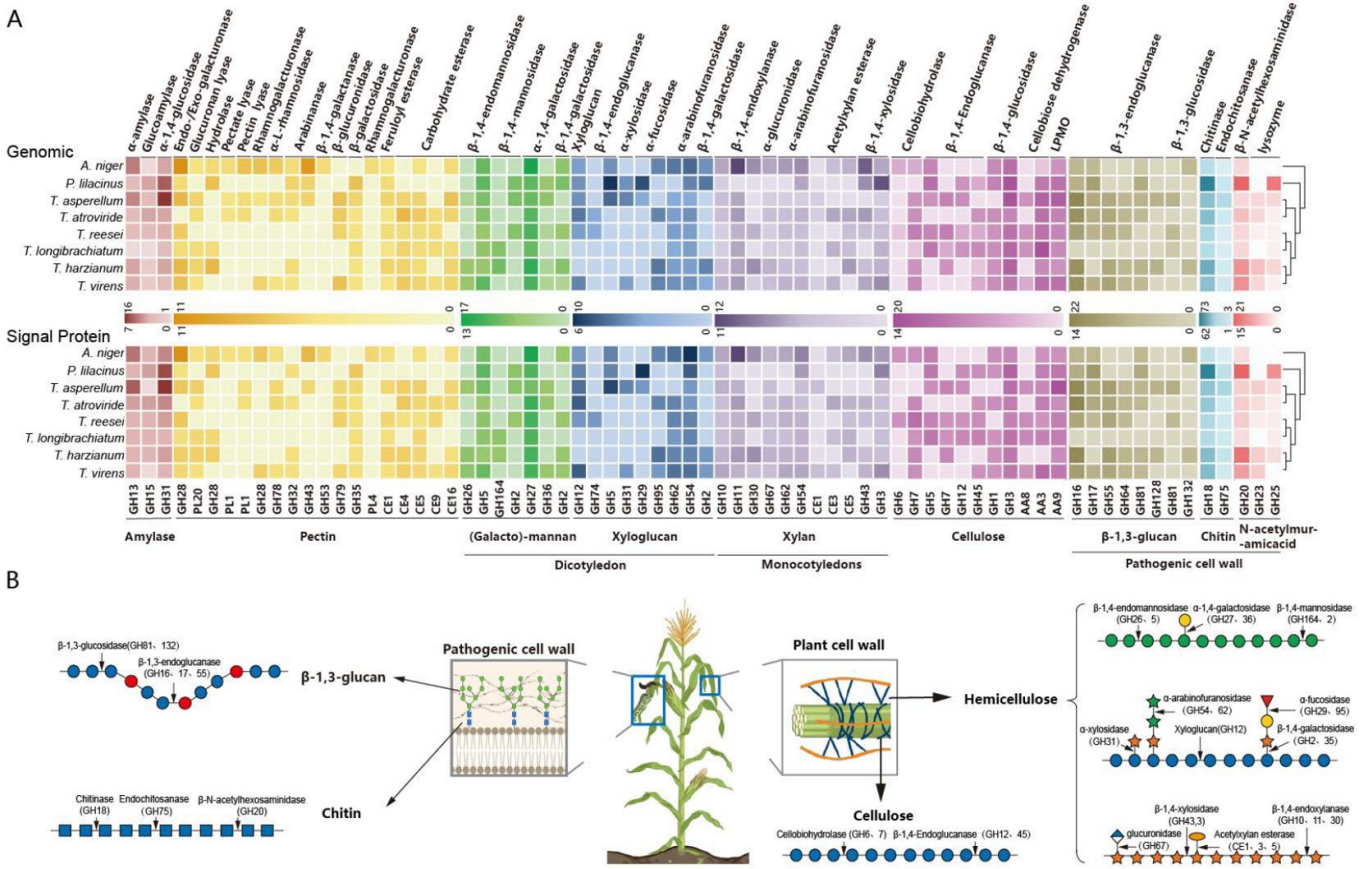
Figure 2 Statistics of CAZymes in the genome and schematic diagram of cell wall degradation in plants and phytopathogenic bacteria (A) Statistics of carbohydrate-active enzyme components in the genomes of eight biocontrol fungi. The number of genomes represents the number of proteases in the genome, while the number of signal proteins represents the number of proteases with signal peptides. (B) Schematic diagram of the composition and degradation of cell walls in plants and phytopathogenic bacteria.

To obtain carbon sources in natural habitats, microorganisms have evolved different carbohydrate-active enzyme (CAZyme) systems [16]. Enzymes involved in carbon source degradation are assigned to at least 35 glycoside hydrolase (GH) families, three carbohydrate esterase (CE) families, and 6 polysaccharide lyase (PL) families [17]. According to the substrate specificity of each family of degradative enzymes, these enzymes can be classified into pectin-, hemicellulose-, or cellulose-degrading enzyme systems or pathogenic organism biodegrading enzymes ( Figure 2) [ 18– 20].

The pathogenic organism biodegrading enzyme system in CAZymes can cleave the cell walls of pathogenic bacteria and the body walls of harmful insects or nematodes to achieve biocontrol effects [21]. Acetylhexosaminidase from the GH20 family and β-1,3-glucosidase from the GH17 family can be used to degrade the cell wall of pathogenic bacteria [20]. The number of acetylhexosaminidases from *P*. *lilacinus* is the greatest, containing a total of 13 genes. The GH18 family contains all fungal chitinases that can invade the cell walls of pathogenic fungi and the eggshell of phytoparasitic nematodes. The number of chitinases reflects the biocontrol potential of different fungi [22]. *P*. *lilacinus* is widely used in biological control because of its high efficiency at controlling nematodes, a property that is associated with the fact that this fungus contains a large number of GH18 family chitinases that cleave the body wall or eggshell of nematodes [23]. Omics data show that *P*. *lilacinus* contains 73 GH18 genes ( Supplementary Table S2). Combined with its efficient globulin degradation mechanism, *P*. *lilacinus* could be used as a predatory biocontrol fungus to effectively destroy the body walls of harmful insects and nematodes and could be directly used at the field level to achieve biocontrol of such pests.

The fungal genus *Trichoderma* contains the largest GH18 chitinase family, and its GH18 family has expanded to varying degrees during the evolution of different species of this genus. *T*. *virens*, *T*. *atrovirens* and *T*. *harzianum* contain 84, 67, and 66 GH18 genes, respectively, whereas *T*. *longibrachiatum* has only 15 GH18 genes ( Supplementary Table S2). *T*. *virens* is a powerful biocontrol fungus [21]. Interestingly, although *Trichoderma* spp. contains genes encoding GH20 and GH18 enzymes, the proteome analysis revealed that few chitinases or β-1,3-glucosidases are produced under exogenous induction conditions. It is speculated that most of these genes in *Trichoderma* are related to the growth of its endogenous cell wall [ 24, 25].

In filamentous fungi, the degradation of lignocellulose is one of the main functions of CAZymes. The distribution of lignocellulose in plants is as follows: pectin and hemicellulose are the main components of the outer layer of plant cell walls, whereas cellulose is wrapped within hemicellulose ( Figure 2B) [16]. Omics data show that the number of endo-/exo-galacturonases that degrade the pectin backbone in plant tissue is increased to 11 in *Aspergillus niger*, which is the highest among the eight filamentous fungi investigated ( Supplementary Table S2). In addition, the *A*. *niger* genome also contains 11 pectin lyases and more than 30 pectin side chain-degrading enzymes [26], which is twice as many as those in *Trichoderma* and *P*. *lilacinus*. Similarly, various hemicellulose-degrading enzymes in *A*. *niger*, such as galactosidases of the GH27 family and endoxylanases of the GH11 family, which are encoded by 17 and 12 genes, respectively, have also undergone gene amplification ( Supplementary Table S2). The large amount of pectin- and hemicellulose-degrading enzymes allows *A*. *niger* to destroy plant cell walls. As a consequence of its phytopathogenic potential, *A*. *niger* cannot be used as a biocontrol fungus [27]. In contrast, *Trichoderma* contains only a very small number of pectin- and hemicellulose-degrading enzymes. The number of cellulose-degrading enzymes in the *Trichoderma* genome is high, with *T*. *longibrachiatum*, *T*. *reesei* and *T*. *asperellum* having the greatest numbers, at 67, 61 and 70, respectively ( Supplementary Table S2). The natural habitat of *Trichoderma* is decomposed wood with only internal cellulose remaining, which directly determines its degradation preferences, with few hemicellulose-degrading enzymes but mostly cellulose-utilizing enzymes [28]. Therefore, it is difficult for *Trichoderma* to break the protective barrier of plant cell walls to cause plant infection, which provides the possibility for members of this genus to be used as biocontrol fungi [29].

## Intracellular secondary Metabolism of Biocontrol Fungi and Characteristics of Their Products

**Figure 3:**
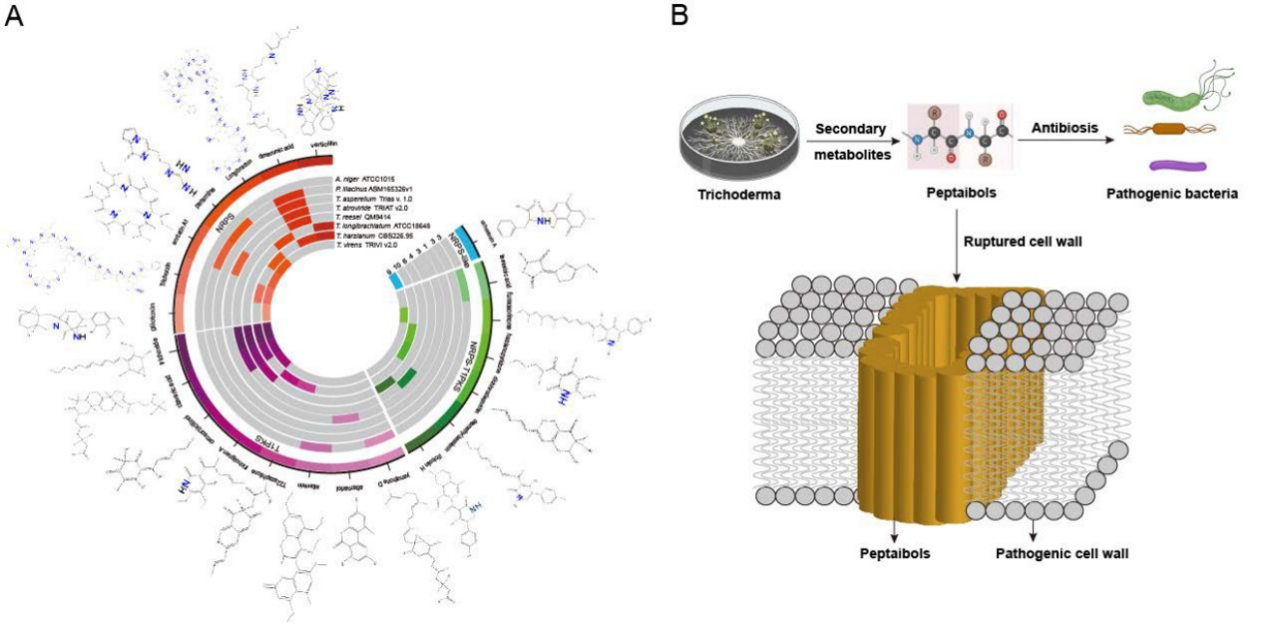
Figure 3 Distribution of secondary metabolites in fungi and mode of action of *Trichoderma* peptaibols (A) The products of the main secondary metabolic pathways in 8 strains of biocontrol fungi. Only secondary metabolites that have been reported to have antibacterial activity were included. (B) Production of peptaibols by Trichoderma and its antibacterial mode of action.

*P*. *lilacinus* mostly relies on extracellular CAZymes and proteases to directly infect pathogenic organisms to achieve biological control, whereas some biocontrol fungi rely on their own secondary metabolites to achieve biocontrol [ 30, 31]. Recently, a range of bioinformatics tools and databases, such as the MIBiG database ( https://mibig.secondarymetabolites.org/), and the antiSMASH ( https://fungismash.secondarymetabolites.org/) and SMURF ( https://www.jcvi.org/) algorithms, have become available for analyzing secondary metabolism-related biosynthesis-related gene clusters (BGCs) and natural products in fungal genomes [8, 32– 34]. The antiSMASH algorithm was used to mine the BGCs of the 8 fungi tested and compare them with those in the MIBIG database to obtain possible second-generation products ( Supplementary Table S3), which could represent the potential value of the different fungi in biological pest and disease control ( Figure 3A).

In filamentous fungi, secondary metabolite BGCs are mainly nonribosomal peptide synthetases (NRPSs) and iterative type 1 polyketide synthases (T1PKSs), and the corresponding products are nonribosomal peptides (NRPs) and polyketides (PKs), respectively. These two products are the most widely studied because they account for the largest number of secondary metabolism BGCs and have special structures [35]. *T*. *harzianum* contains 86 secondary metabolite BGCs and is the most widely used biocontrol agent in the genus *Trichoderma* [36]. By comparison, *A*. *niger* and *P*. *lilacinus* both contain only three secondary metabolites with reported antibacterial activity, whereas *T*. *harzianum*, *T*. *virens* and *T*. *longibrachiatum* contain 10, 9 and 6, respectively, the greatest numbers of which are among the 8 fungi ( Figure 3A). Therefore, *Trichoderma* is often used as a biocontrol fungus, and antibiosis is used in the field to effectively inhibit pathogenic bacteria [37].

The special structure of metabolites is mostly related to precursor substances. The precursors of NRPs and PKs are the various amino acids produced by protease degradation of proteins and the oligosaccharides produced by the degradation of carbon sources by CAZymes, respectively [ 38, 39]. Precisely because of the difference in precursor substances, the atomic composition of the secondary metabolites reveal that all the NRPs contain high concentrations of nitrogen, whereas the PKs contain a larger proportion of carbon ( Figure 3A). This atomic composition characteristic indicates that the synthesis of antibiotics can only be completed when extracellular carbon and nitrogen biomass can be continuously supplied to the biocontrol agent, thereby inhibiting phytopathogenic organisms and achieving the intended biocontrol purpose.

In addition to their own atomic composition characteristics, secondary metabolites have been extensively studied for their antibacterial activity in biocontrol fungi. Typical peptide products in NRPs are peptaibols composed of up to 20 amino acids, most of which are found in species of the *Trichoderma* genus [40]. Among the 7 NRPs identified in the database ( Figure 3A), 6 are peptaibols, and 1 is a gliotoxin peptide [41]. Both *T*. *harzianum* and *T*. *virens* secrete an 18-amino acid peptaibol called trichorzin, a peptide antibiotic. This peptide has the polar C-terminus of Trp and has affinity for the hydrophilic head of the phospholipid molecules in the bilayer membrane, which is very important for building ion channels in the lipid bilayer membrane to disrupt the ion balance of the cell ( Figure 3B) [42]. *T*. *longibrachiatum* secretes a unique peptaibol called longibrachin (LG), which consists of a 20-amino acid residue. It not only shows antibacterial activity against *Mycoplasma* and gram-positive bacteria but also displays weak inhibitory effects against the human pathogenic fungus *Aspergillus fumigatus* [ 43, 44]. In addition to synthesizing peptaibols, *T*. *virens* also encodes the gliotoxin peptide, the activity of which is believed to be related to the disulfide bond that generates reactive oxygen species and inactivates proteins through covalent modification of thiol groups [ 45, 46].

Unlike NRPs, PKs mostly have unique siderophore activity. Among the 8 filamentous fungi, only *T*. *harzianum* secretes tricholignan A ( Figure 3A), which exhibits siderophore activity. Under iron-deficient conditions, this redox-active trisubstituted *o*-hydroquinone can reduce Fe(III) to Fe(II) and alleviate the iron deficiency phenotype of the model plant *Arabidopsis*. This compound was previously characterized in the biofertilizer *T*. *harzianum* strain t-22 [47].

## Conversion Relationship between Extracellular Primary Metabolism and Intracellular Secondary Metabolism in Biocontrol Fungi

**Figure 4:**
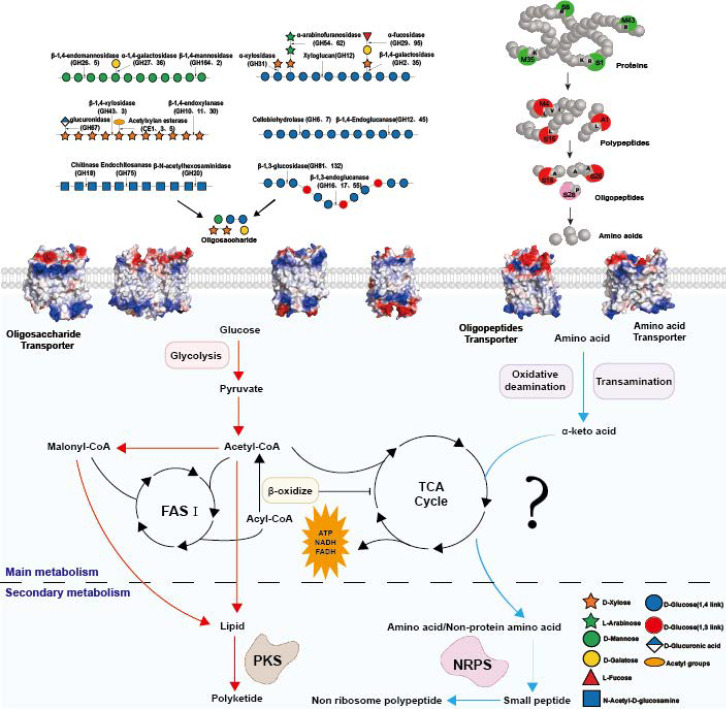
Figure 4 Metabolic pathways involved in the transition between extracellular primary metabolism and intracellular secondary metabolism

CAZymes and proteases in the extracellular primary metabolism of biocontrol fungi can cleave the protective barrier of target phytopathogenic organisms to achieve biocontrol effects. The degradation of carbon and nitrogen biomass in the environment into absorbable small organic molecules to continuously provide precursor substances for intracellular secondary metabolism is an important function [48]. Therefore, by reviewing the conversion relationship between the substrate-specific preference of extracellular hydrolases and intracellular secondary metabolites, it is crucial to ensure that biocontrol fungi have a continuous supply of carbon and nitrogen for the synthesis of secondary metabolites ( Figure 4).

In recent years, research teams have verified several metabolic conversion relationships through omics, thereby improving the production of secondary metabolites [ 49, 50]. Figure 4 summarizes the relevant conversion pathways. PKs are formed by continuously condensing carbon source degradation products (malonyl coenzyme A (malonyl-CoA, acetyl-CoA)) into the β-polyketide skeleton through T1PKS and then cyclizing it into polycyclic compounds [38]. The main source pathways of its predominant precursor substance, acetyl-CoA [51], include glycolysis and lipid oxidation, with lipid oxidation being mostly observed in bacteria and the yeast *Yarrowia lipolytica* [ 2, 50]. The carbon skeleton involved in glycolysis in filamentous fungi is mostly derived from various oligosaccharides produced by the degradation of polysaccharides by extracellular CAZymes. Compared with other filamentous fungi, Trichoderma contains more CAZymes, especially cellulases, which can efficiently degrade cellulose into oligosaccharides, thus providing a large amount of carbon skeletons for the synthesis of PKs.

Compared with that of PKs, the synthesis of NRPs involves the continuous condensation of amino acids and nonprotein amino acids through NRPSs to assemble into specific peptide products. The supply of precursor substrates for NRPs mostly relies on the hydrolysis of extracellular proteases [39]. Extracellular peptides are degraded into amino acids under the action of proteases. After amino acids enter fungal cells through transporters, they undergo a series of transamination reactions to generate α-keto acids, which subsequently enter the tricarboxylic acid (TCA) cycle. The intermediate products of the TCA cycle generate additional amino acids or nonprotein amino acids as precursors from which to synthesize NRPs. As a highly efficient protein degrader, *P*. *lilacinus* itself contains few NRP synthesis genes, but in mixed bacterial agents, it can degrade globulin present in the environment into oligopeptides for use by other antibiotic-producing biocontrol fungi. In contrast, although *Trichoderma* has a large number of NRPS genes, its ability to specifically degrade globulin in nematodes in natural habitats is weak due to a relative lack of precursor substrates. Therefore, prior to its application in the field, *Trichoderma* is often compounded with organic fertilizers rich in small peptides [52].

In addition to directly increasing the supply of precursor substances by analyzing the substrate preferences of different biocontrol fungi, analyzing the transport specificity of transporters through structural proteomics can also indirectly increase the intracellular content of precursor substances. Oligosaccharide transporters and amino acid and oligopeptide transporters are mostly major facilitator superfamily (MFS) transporters, with lengths ranging from 400 to 600 amino acids. There is a nonnegligible correlation between the transport efficiency of transporters and their surface potential [53]. Transport specificity is usually manifested by the fact that when a transport protein has more than 70% negatively charged residues (acidic amino acids) on the extracellular side, it can better bind to substrates with positive charges. Conversely, when the transport protein has more than 63% positively charged residues (basic amino acids) on the extracellular side, the substrate is mostly negatively charged. Understanding substrate-specific transport mechanisms on the basis of structural proteomics provides a theoretical basis for improving transport efficiency by modifying surface properties [ 54– 56].

## Concluding Remarks and Future Perspectives

Biological control plays an increasingly important role in pest and pathogen control in crops due to its environmental sustainability and low cost [57]. Among the various microorganisms used in biocontrol practices, filamentous fungi, such as *Paecilomyces* and *Trichoderma*, which are common in soil, have been widely studied, with *Trichoderma* accounting for 60% of registered biocontrol agents [58]. The effect of applying a filamentous fungus to the field is closely related to its biocontrol classification. Filamentous fungi can achieve antibacterial effects by secreting various bioactive substances. According to the different antibacterial mechanisms exhibited by the bioactive substances they secrete, biocontrol agents can be divided into predatory and antibiosis types. The antibacterial mechanism of predatory biocontrol fungi involves the secretion of various free enzymes, such as β-1,3-glucosidase, GH18, and protease, to directly degrade pathogenic bacteria. Antibiotic biocontrol fungi have a large number of secondary metabolite synthesis gene clusters, which can inhibit the growth of pathogenic bacteria by synthesizing secondary metabolites such as NRPs and PKs. This type of biocontrol fungus relies on CAZymes and proteases to continuously generate secondary metabolite precursors in the field. However, due to the current lack of clarity regarding the mechanisms by which different filamentous fungi inhibit pathogens, the application of these fungi to the field faces challenges such as the inability to establish dominant strains and the limitations of secondary metabolism. Therefore, understanding the specific intracellular and extracellular metabolite preferences of filamentous fungi is crucial for determining their mode of biocontrol in their natural ecological niche.

With the continuous development of omics, omics big data generated from genomics and structural proteomics have provided new ideas for in-depth exploration of different modes of biocontrol by filamentous fungi. By comparing the annotation information on CAZymes and proteases in the genome ( Figures 1 and 2), *P*. *lilacinus* was shown to contain 4 times more proteases than each of the other 7 filamentous fungi tested. Moreover, the genes encoding S8 family proteases have undergone significant amplification during evolution, giving *P*. *lilacinus* the ability to rapidly cleave the cell wall of pathogenic bacteria; at the same time, most of its CAZymes are enzymes that lyse pathogen protective barriers, such as GH18, GH20 and β-1,3-glucosidase. Therefore, *P*. *lilacinus* can be used directly in the field as a typical predatory biocontrol fungus to effectively reduce plant-pathogenic diseases caused by bacteria and nematodes. *A*. *niger* prefers hemicelluloses, such as pectin and xylan, as carbon sources, causing plant cell wall rupture, so it often appears to be a phytopathogen. Because its natural habitat is partially decomposed wood, *Trichoderma* has downregulated its ability to degrade pectin and hemicellulose during the evolutionary process. It prefers cellulose as a carbon source and does not damage plant cell walls to cause crop disease.

Further in-depth exploration of secondary metabolism biosynthesis-related gene clusters and metabolite characteristics ( Figure 3) and comprehensive analysis of metabolite transformation pathways recorded in the literature, combined with transporter structural omics ( Figure 4), revealed that *Trichoderma* contains the largest number of PKs and NRPs. Therefore, *Trichoderma* can be widely used as a biocontrol fungus via antibiosis while ensuring that it does not damage plants. In addition, because *Trichoderma* lacks the S8 family serine protease to degrade globulin and other proteases that specifically hydrolyze hydrophobic amino acids (Leu, Ile, Val, Ala, etc.), it is unable to continuously provide amino acid precursors for the synthesis of NRPs. Analysis of the metabolic conversion relationship revealed that this problem can be solved in practical applications by compounding the biocontrol agent with organic fertilizers rich in peptides. However, there is still little research on the relationship between the surface charge of filamentous fungal transporters and substrates, as well as intracellular and extracellular metabolic conversion, especially the relationship between NRPs and nitrogen source conversion. Therefore, in future work, how to analyze the intracellular and extracellular metabolism of biocontrol fungi more quantitatively and at higher levels through metabolomics, transcriptomics, proteomics and other omics technologies will be an important direction for further refining the different types of fungal biological control and formulating strategies for optimizing the ability of biocontrol agents to control crop pests and pathogens.

## Supporting information

603TableS1-3

## Supplementary Data

Supplementary data is available at *Acta Biochimica et Biphysica Sinica* online.
