# Supplementary material for: Unraveling the metabolic potential of biocontrol fungi through omics data: a key to enhancing large-scaleapplication strategies: Omics data unveils biocontrol fungi’s metabolic potential
Source: Acta Biochim Biophys Sin (Shanghai). 2024 Apr 29;56(6):825–32. doi: 10.3724/abbs.2024056 (PMC11214957; doi:10.3724/abbs.2024056)
Supplement: 603TableS1-3 [file 603TableS1-3.pdf]

Supplementary Table S1. Statistics of endopeptidases in the genomes of 8 biocontrol fungi

Enzyme type		Protein family	*A. niger* ATCC1015		*P. lilacinus* ASM165326v1		*T. asperellum* Trias v. 1.0		*T. atroviride* TRIAT v2.0		*T. reesei* QM9414		*T. longibrachiatum* ATCC18648		*T. harzianum* CBS226.95		*T. virens* TRIVI v2.0	
			Signal P.	Genome	Signal P.	Genome	Signal P.	Genome	Signal P.	Genome	Signal P.	Genome	Signal P.	Genome	Signal P.	Genome	Signal P.	Genome
Endopeptidases	Serine endopeptidases	S1	0	0	4	14	0	0	0	0	1	1	1	2	0	1	0	0
		S1C	1	1	0	5	0	0	0	0	0	1	0	1	1	1	0	0
		S8	2	10	48	77	0	0	0	1	0	0	7	13	8	33	0	1
		S9	0	1	1	6	0	0	0	0	0	0	0	3	0	0	0	0
		S14	0	1	0	1	0	1	0	1	0	1	0	1	0	1	0	1
		S16	0	2	0	5	0	4	1	2	0	2	0	2	1	2	1	2
		S26	0	1	1	3	0	0	0	0	0	1	0	1	1	1	0	0
		S26B	1	1	0	9	0	5	1	4	0	1	0	1	1	1	0	1
		S28	1	2	5	5	1	3	0	0	8	15	0	1	7	12	0	1
		S53	1	6	7	8	0	0	0	0	0	0	0	0	2	11	0	0
		S54	0	1	0	1	0	2	0	1	0	1	0	1	1	1	0	1
	Cysteine endo peptidases	C2	0	2	0	8	0	2	0	0	0	3	0	3	0	3	0	0
		C13	0	0	2	2	0	0	0	0	0	0	0	0	0	0	0	0
		C14B	1	4	0	5	0	0	0	0	0	2	0	2	1	2	0	0
		C15	0	1	0	3	0	2	0	1	0	1	0	1	0	1	0	1
		C40	0	0	3	3	0	0	0	0	0	0	0	0	0	0	0	0
		C45	0	0	0	2	0	0	0	0	0	0	0	0	0	0	0	0
		C48	0	2	0	0	0	0	0	0	0	3	0	2	0	4	0	0
		C50	0	0	0	2	0	2	0	1	0	0	0	1	0	0	0	1
		C51	0	0	0	0	0	0	0	0	0	0	0	0	0	1	0	0
		C54	1	1	0	2	0	1	1	1	0	1	0	1	0	1	0	1
		C58	0	0	0	1	0	0	0	0	0	0	0	0	0	0	0	0
		M76	0	0	0	2	0	0	0	0	0	1	0	0	0	0	0	0
	Aspartic endopeptidases	A1	6	18	33	43	1	3	0	0	7	10	9	13	8	27	0	0
		A2	0	0	0	1	0	0	0	0	0	0	0	0	0	3	0	0
		A4	0	0	5	7	0	0	0	0	0	0	0	0	0	0	0	0
		A22B	1	1	0	2	0	3	0	1	0	0	0	1	0	1	0	2
	Metalloend peptidases	M3	0	4	0	14	0	7	1	4	0	2	0	2	3	5	0	5
		M4	0	1	0	3	0	0	0	0	1	2	0	2	1	2	0	0
		M6	0	0	0	0	0	0	0	0	0	0	0	0	1	1	0	0
		M10	0	0	0	1	0	0	0	0	0	0	0	0	0	0	0	0
		M11	0	0	3	3	0	0	0	0	0	0	0	0	0	0	0	0
		M12	0	0	0	1	0	0	0	0	0	0	0	0	0	0	0	0
		M16	0	3	0	10	0	4	0	2	0	2	0	2	0	0	0	2

		M28B	1	2	0	6	0	0	0	0	0	0	0	0	0	0	0	0
		M35	0	0	14	21	2	2	0	1	0	0	0	0	0	1	1	1
		M36	0	1	5	6	3	4	0	1	1	1	1	1	0	1	0	1
		M41	0	2	2	5	0	0	0	0	0	0	0	3	1	2	0	0
		M43B	0	1	9	18	0	1	0	2	0	2	0	1	2	2	0	1
		M48	0	1	0	2	0	1	0	0	0	0	0	1	0	0	0	0
		M60	0	0	1	1	0	0	0	0	0	0	0	0	0	0	0	0
		M66	0	0	0	1	0	0	0	0	0	0	0	0	0	0	0	0
		M67	0	5	0	7	0	0	0	0	0	8	1	4	0	4	0	0
		M48A	1	1	2	5	0	3	0	1	0	2	0	2	0	1	0	1
	Total		17	76	145	321	7	50	4	24	18	63	19	68	39	126	2	23

Supplementary Table S2. Statistics of CAZymes components in the genomes of 8 biocontrol fungi

Substrate	Enzyme activity	CAZy family	*A. niger* ATCC1015		*P. lilacinus* ASM165326v1		*T. asperellum* Trias v. 1.0		*T. atroviride* TRIAT v2.0		*T. reesei* QM9414		*T. longibrachiatum* ATCC18648		*T. harzianum* CBS226.95		*T. virens* TRIVI v2.0	
			Signal P.	Genome	Signal P.	Genome	Signal P.	Genome	Signal P.	Genome	Signal P.	Genome	Signal P.	Genome	Signal P.	Genome	Signal P.	Genome
Amylase	α-amylase	GH13	4	9	1	3	5	8	2	3	1	3	1	1	1	5	1	4
	Glucoamylase	GH15	1	1	2	5	0	3	2	5	1	3	1	1	1	2	0	2
	α-1,4-glucosidase	GH31	3	5	6	12	7	16	3	5	2	4	1	3	2	5	1	3
Pectin	Endo-/Exo-galacturonase	GH28	11	11	1	2	6	6	0	0	4	4	1	2	3	6	2	5
	Glucuronan lyase	PL20	1	1	0	0	3	3	1	2	0	0	2	2	3	3	1	1
	hydrolase	GH28	2	2	4	5	0	0	0	0	0	0	2	2	3	6	0	0
	Pectate lyase	PL1	1	2	0	0	0	1	0	1	0	0	0	0	0	0	0	0
	Pectin lyase	PL1	1	5	0	0	1	2	1	2	0	0	0	0	0	0	0	0
	Rhamnogalacturonase	GH28	4	5	0	0	0	1	0	0	0	0	0	0	0	0	3	3
	α-L-rhamnosidase	GH78	1	4	0	0	2	2	2	2	0	0	0	0	0	0	1	1
	Arabinanase	GH32	0	2	1	3	0	2	1	1	0	0	0	0	1	2	1	1
		GH43	6	10	0	3	2	3	0	0	0	0	0	0	0	0	0	0
	β-1,4-galactanase	GH53	2	2	0	0	0	0	0	0	0	0	0	0	0	0	0	0
	β-glucuronidase	GH79	0	0	0	0	2	5	2	4	3	3	0	0	0	0	2	5
	β-galactosidase	GH35	0	0	4	5	2	3	1	1	1	1	1	2	1	1	1	1
	Feruloyl esterase	CE1	1	1	0	2	2	3	0	1	2	2	1	1	3	3	1	1
	Carbohydrate esterase	CE4	0	0	0	0	3	3	4	6	1	2	0	3	3	3	3	4
		CE5	1	1	0	0	3	3	2	4	1	2	2	2	2	2	2	3
		CE9	0	0	0	0	0	0	2	2	2	2	0	2	0	0	1	2
		CE16	1	2	0	0	2	5	2	3	0	0	0	2	1	3	2	3
Galacto) -	β-1,4-endomannosidase	GH26	0	0	0	0	1	1	0	0	0	0	0	0	2	2	2	2

**mannan**		**GH5**	4	7	6	8	4	5	1	4	2	2	2	3	1	2	5	8
	**β-1,4-mannosidase**	**GH164**	0	0	0	0	0	0	0	0	0	0	2	4	1	6	0	0
		**GH2**	0	0	2	5	2	5	0	0	1	4	0	0	0	0	0	0
	**α-1,4-galactosidase**	**GH27**	13	17	7	8	4	7	9	9	8	10	7	7	8	13	8	10
		**GH36**	0	0	0	2	1	3	1	0	0	1	0	0	0	1	1	1
	**β-1,4-galactosidase**	**GH2**	1	3	0	5	0	0	0	0	1	2	0	0	1	4	1	1
	**Xyloglucan**	**GH12**	1	2	2	3	3	3	4	5	1	2	0	0	0	0	4	4
	**β-1,4-endoglucanase**	**GH74**	0	0	0	0	0	0	0	1	1	1	0	0	0	0	0	0
		**GH5**	2	3	2	10	5	6	0	0	0	0	0	0	0	0	0	0
	**α-xylosidase**	**GH31**	0	0	0	3	2	4	0	0	0	0	0	0	0	0	1	2
**Xyloglucan**	**α-fucosidase**	**GH29**	0	0	6	8	1	1	0	0	0	0	0	0	0	0	0	0
		**GH95**	2	4	0	0	0	0	2	4	0	0	0	0	3	5	1	4
	**α-arabinofuranosidase**	**GH62**	3	5	0	0	0	1	2	2	2	2	1	1	2	2	3	3
		**GH54**	6	6	4	4	1	1	1	3	2	2	2	2	2	2	3	3
	**β-1,4-galactosidase**	**GH2**	1	3	0	5	0	0	0	0	0	0	0	0	1	4	1	1
		**GH10**	3	4	1	1	2	2	1	2	1	1	1	1	2	2	3	3
	**β-1,4-endoxylanase**	**GH11**	11	12	0	0	2	4	4	5	2	3	2	4	4	4	2	5
		**GH30**	5	6	0	0	0	0	0	0	3	3	0	0	5	6	1	1
	**α-glucuronidase**	**GH67**	2	4	0	0	1	1	1	2	1	1	1	1	2	3	1	2
	**α-arabinofuranosidase**	**GH62**	3	5	0	0	0	1	2	2	2	2	1	1	2	2	3	3
**Xylan**		**GH54**	6	6	4	4	1	1	1	3	2	2	2	2	2	2	3	3
		**CE1**	0	0	0	0	1	1	0	1	0	0	0	0	0	0	1	1
	**Acetylxylan esterase**	**CE3**	1	2	0	0	0	0	3	4	0	0	1	3	0	0	1	3
		**CE5**	3	3	0	0	0	0	2	4	1	2	2	2	2	5	0	0
	**β-1,4-xylosidase**	**GH43**	5	10	0	6	0	0	2	5	1	2	0	0	1	2	2	4
		**GH3**	4	4	6	11	1	1	1	2	1	2	1	3	1	3	5	6
	**Cellobiohydrolase (CBH)**	**GH6**	1	2	0	0	0	0	0	0	1	1	0	0	0	0	0	0
		**GH7**	2	2	0	0	0	3	3	4	8	8	1	1	3	3	2	5
		**GH5**	5	6	6	12	2	3	0	0	4	6	6	10	0	0	0	0
	**β-1,4-Endoglucanase**	**GH7**	0	0	0	0	4	7	0	0	1	8	1	2	3	3	0	0
		**GH12**	0	0	2	3	2	4	0	0	1	2	3	3	0	0	0	0
**Cellulose**		**GH45**	0	0	2	2	0	0	1	3	2	2	4	5	0	1	1	2
	**β-1,4-glucosidase**	**GH1**	2	5	1	7	0	0	2	4	0	3	1	4	1	3	1	4
		**GH3**	3	12	10	19	8	19	5	6	4	11	7	14	6	15	6	10
	**Cellobiose dehydrogenase**	**AA8**	0	0	0	0	2	3	0	0	2	3	3	3	0	0	1	2
	**(CDH)**	**AA3**	1	1	5	9	3	15	5	11	1	11	4	20	0	5	9	12
	**LPMO**	**AA9**	1	3	6	11	14	16	2	7	5	6	5	5	2	3	6	11
**Pathogenic cell wall**		**GH16**	2	3	4	5	13	22	14	18	6	12	0	0	13	18	8	18
	**β-1,3-endoglucanase**	**GH17**	2	2	9	12	6	8	5	6	1	4	0	0	0	0	3	6
		**GH55**	0	0	0	0	8	8	6	9	6	6	0	0	8	9	6	11

	GH64	0	0	0	0	2	3	0	0	1	3	0	0	2	3	2	3
	GH81	1	1	5	7	2	4	0	0	0	0	0	0	2	4	1	0
	GH128	0	0	0	0	3	5	0	0	0	0	0	0	2	5	0	0
β-1,3-glucosidase	GH81	0	0	0	0	4	5	0	0	1	2	0	1	0	0	1	2
	GH132	2	2	0	0	1	2	0	0	0	0	0	0	1	2	0	0
Chitinase	GH18	6	14	62	73	23	34	21	36	11	19	9	15	28	47	28	34
Endochitosanase	GH75	1	2	6	8	12	16	3	6	3	3	3	3	5	5	3	6
β-N-acetylhexosaminidase	GH20	2	3	10	13	3	3	3	5	3	3	2	3	3	6	2	3
lysozyme	GH23	0	0	0	0	3	3	2	3	1	1	0	0	3	5	3	3
	GH25	0	0	7	11	2	3	1	1	1	1	1	1	2	3	0	1

Supplementary Table S3. Putative biosynthetic gene clusters (BGCs) coding for secondary metabolites and their products from 8 strains of biocontrol fungi

Organism	Region	Similarity	Type	Compound
A. niger ATCC1015	4.1	75%	NRPS	nidulanin A
	4.2	75%	T1PKS-NRPS	terrestric acid
	5.1	87%	T1PKS	pyranoviolin A
	5.2	87%	T1PKS	burnettiene A
	6.2	100%	T1PKS	pyranonigrin E
	7.1	60%	T1PKS	aflavarin
	10.5	100%	NRPS	serinocyclin
	13.1	100%	T1PKS	yanuthone D
	22.3	100%	NRPS,T1PKS	AbT1
	23.3	100%	T1PKS,NRPS-Like	pyrophen
	24.3	100%	NRPS,T1PKS	azanigerone C
	24.5	66%	NRPS-Like	kojic acid
	24.7	100%	T1PKS	naphthopyrones

P. lilacinus ASM165326v1	3.1	100%	NRPS	enniatin A1
	4.2	95%	T1PKS,NRPS	leucinostatin
	4.3	75%	NRPS	dimerumic acid
	5.4	100%	NRPS	peramine
T. asperellum Trias v. 1.0	1.2	100%	NRPS-Like	choline
	6.1	62%	NRPS	dimerumic acid
T. atroviride TRIAT v2.0	3.1	100%	NRPS	enniatin A1
	3.4	100%	NRPS-Like	choline
	7.1	62%	NRPS	dimerumic acid
	7.4	100%	T1PKS	alternariol
T. reesei QM9414	1.2	100%	T1PKS	clavaric acid
	1.4	60%	NRPS-T1PKS	desmethyl bassianin
	4.2	62%	NRPS	dimerumic acid
	6.2	66%	T1PKS	trichoxide
	6.4	81%	NRPS	melinacidin IV
	6.6	100%	NRPS-Like	choline
T. longibrachiatum ATCC18648	1.1	100%	NRPS	longibrachin
	1.3	84%	NRPS	verticillin
	1.4	66%	T1PKS	trichoxide
	4.2	100%	T1PKS	clavaric acid
	5.1	75%	T1PKS	oxosorbicillinol
	9.3	100%	T1PKS	naphthopyrones
	35.1	100%	T1PKS-NRPS	ilicicolin H
T. harzianum CBS226.95	2.1	100%	NRPS	trichorzin
	3.2	100%	T1PKS	clavaric acid
	4.3	100%	NRPS	peramine
	8.1	84%	NRPS	verticillin
	8.4	83%	T1PKS	trichoxide
	10.2	66%	NRPS-T1PKS	dichlorodiaporthin
	15.1	75%	NRPS	dimerumic acid
	16.1	100%	NRPS-T1PKS	harzianopyridone
	21.2	100%	T1PKS	tricholignan A
	27.1	80%	T1PKS	T22azaphilone
T. virens	2.2	100%	NRPS	trichorzin

TRIVI v2.0	8.1	66%	T1PKS	trichoxide
	8.2	75%	T1PKA	oxosorbicillinol
	12.1	100%	NRPS	enniatin A1
	13.1	100%	T1PKS	clavaric acid
	15.1	66%	NRPS-T1PKS	fumosorinone
	16.1	100%	NRPS-Like	ochratoxin A
	47.1	100%	NRPS	gliotoxin
	91.3	100%	NRPS	peramine
